# Reproducible and stable cycling performance data on secondary zinc oxygen batteries

**DOI:** 10.1038/s41597-020-00728-3

**Published:** 2020-11-13

**Authors:** Saustin Dongmo, Julian Jakob Alexander Kreissl, Kohei Miyazaki, Takeshi Abe, Ting-Hsuan You, Chi-Chang Hu, Daniel Schröder

**Affiliations:** 1 Institute of Physical Chemistry, Justus-Liebig-University Giessen, Heinrich-Buff-Ring 17, D-35392 Giessen, Germany; 2 Center for Materials Research (LaMa), Justus-Liebig-University Giessen, Heinrich-Buff-Ring 16, D-35392 Giessen, Germany; 3 Department of Energy & Hydrocarbon Chemistry, Kyoto University, Nishikyo-ku, 615-8510 Kyoto Japan; 4 Laboratory of Electrochemistry & Advanced Materials, Department of Chemical Engineering, National Tsing-Hua University, Kuang-Fu Road, 30013 Hsinchu, Taiwan

**Keywords:** Batteries, Energy

## Abstract

Electrically rechargeable zinc oxygen batteries are promising energy storage devices. They appeal due to the abundance of zinc metal and their high energy density. Research on zinc oxygen batteries is currently focusing on the development of electrode materials. Since the progress is rapid and no state-of-the-art is agreed upon yet, it is difficult to benchmark their performance. This circumstance also complicates the use of the generated electrochemical data for model-based research – simulating the processes in the battery requires reliable performance data and material properties from experimental investigations. Herein we describe reproducible data on the cycling performance and durability of zinc oxygen batteries. We utilize anodes and gas diffusion electrodes (with the bifunctional catalysts Sr_2_CoO_3_Cl, Ru-Sn oxide, and Fe_0.1_Ni_0.9_Co_2_O_4_ with activated carbon) with low degradation during cycling, and present voltage data of current-dependent discharge and charge. All in all, we stimulate to reuse the data for parameter fitting in model-based work, and also to evaluate novel battery materials by preventing or minimizing side reactions with the testing protocol and setup utilized.

## Abstract (summary)

**Table:** 

Measurement(s)	battery cycling performance • Voltage • Electrical Current • cycling stability
Technology Type(s)	galvanostat • electrochemical analysis

Machine-accessible metadata file describing the reported data: 10.6084/m9.figshare.13066070

## Background & Summary

The zinc oxygen battery (ZOB) could be a promising candidate to power mobile and wearable electronic devices due to its advantages such as low cost, minimal or no ecological problems and safety during operation compared to the lithium-based battery technology^1,2^. The abundance of zinc (Zn) metal makes the ZOB one possible option for the next generation of energy storage devices^1–3^.

Moreover, the ZOB appeals as a simple and practical example to test novel catalysts for the oxygen reduction reaction (ORR) and/or the oxygen evolution reaction (OER) in lab-scale research. Thereby, research on ZOBs with different home-made cell setups should contain precise information on the cell assembly, the volume of electrolyte and the mass of the active material in the electrodes to ensure that reproducible data can be obtained^4,5^. Many parameters – such as the catalyst loading on the cathode gas diffusion layer as well as the gas diffusion layer structure (thickness, porosity and composition) or the composition of the gas used – can influence the cell performance and the long-term stability. A wide range of experimental test parameters has been used in different studies, with different environmental conditions such as temperature, humidity and CO_2_ concentration^4^. Only a small number of studies actually state the information and data necessary to assess the cycling performance and the durability of ZOBs with novel materials^5^. The differences in reported values make it much more difficult to reproduce and compare performance data for ZOBs in comparison to the lithium ion battery. For the latter technolgy, the use of standard cells and same experimental conditions allows researchers to reduce the sources of variability and hence improve the reproducibility of the results^6^. Next to pouch cells, the coin-type cell is now recognized as a suitable standard cell to benchmark novel materials and electrolytes for lithium ion batteries^7^.

The technology of primary (single discharge only) ZOB coin-type cells is quite advanced and already available on the market for various applications in small electronic devices. Bonnick et al. comment on the coin-type cell as a suitable cell for the electrically rechargeable alkaline nickel-zinc battery, which could be extended to the concept of electrically rechargeable ZOB^6^. Some research groups attempted to use the coin-type cells to test new electrodes for ZOBs but the performance data reported were not primarily aimed at the long-term stable use^8,9^.

In this work, we describe data of ZOBs that was obtained by utilizing good performing anode and cathode materials with high stability. Extending the work by Lao-atiman et al. on the discharge only^10^, we describe reproducible experimental data of the long-term stable cycling, i.e. the repeated discharge/charge, for zinc oxygen batteries with three types of bifunctional catalyst at the cathode^11–13^. The voltage data for discharge and charge (provided at various state-of-charge and current density additionally) as well as the reproducibility of the measurements are highly appealing to be used in model-based research. We want to stimulate others to reuse the data for parameter fitting in model-based work as well as to evaluate novel battery materials by the testing setups used. In the end, data for a multitude of different battery types, geometries for electrodes or separators can be generated for future research in the field of energy storage.

## Methods

### Electrode preparation

Zn foil anode (2 mm thickness, >99.99%, ChemPur) was polished before use with P4000 SiC paper (5 µm; Buehler) the preparation of Zn sponge anodes and gas diffusion layer with Sr_2_CoO_3_Cl, Ru-Sn oxide (here 70 atom-% RuO_2_ and 30 atom-% SnO_2_, denoted as RuSn73) or Fe_0.1_Ni_0.9_Co_2_O_4_ (with different amount of activated carbon (AC) as conductive additive) as catalyst, i.e. referred to gas diffusion electrode (GDE) in the following, was carried out as described previously^11–13^. The three different procedures for the preparation of the cathodes are briefly summarized in the following.

Cathode 1^11^: A dispersion was prepared by mixing 0.450 g Sr_2_CoO_3_Cl catalyst^14^, 0.450 g Vulcan XC-72 (Cabot Corporation) and 0.166 g PTFE (60% solution, Sigma Aldrich) in proportion 45:45:10 wt% in 1:1 solution of N-methylpyrrolidone and water (by weight). After stirring, the dispersion was sprayed on carbon paper (PTFE-treated, Toray) and dried under vacuum at 80 °C over night. To ensure good electronic contact to the catalyst, the samples were pressed (9.8 kN cm^−2^ for 1 min) and annealed for 20 min at 375 °C. The resulting GDE (10 mm diameter) was loaded with 2.7 mg cm^−2^ Sr_2_CoO_3_Cl.

Cathode 2^12^: A homogeneous mixture containing 0.100 g (Ru-Sn)O_2_ powder, 0.250 g ethylene glycol, and 0.035 g Nafion® was prepared. This paste was coated onto the carbon paper to form a GDE sheet with an exposed surface area of 10 mm × 10 mm for the electrocatalysts by means of the doctor-blade method without any pressing. Finally, the GDEs were dried in an oven at 85 °C for 24 h.

Cathode 3^13^: The air cathode was fabricated by coating each catalyst (e.g. Fe_0.1_Ni_0.9_Co_2_O_4_/3.7 wt%AC) onto one piece of carbon paper (GDL240 by CeTech Co., Ltd., Taiwan). 0.10 g catalyst powder, 0.10 g ethylene glycol and 0.02 g 5% Nafion were mixed to obtain a homogeneous paste. Then, the paste was evenly coated on the carbon paper without any metallic current collector, and dried overnight at 80 °C. The paste was coated onto 1.0 cm^2^ of the carbon paper.

### Assembly of zinc oxygen cells and operation

Stainless steel CR2032 coin-type cells (TOB New Energy Limited) were prepared using the following assembly protocol: the freshly polished Zn foil (12 mm diameter) anode was placed on top of a tin (Sn) disk current collector (16 mm diameter for CR2032 housing, >99.99%, Chempur) and covered with 150 µL of 4 mol dm^−3^ KOH aqueous electrolyte. Four laminated (16 mm diameter for CR2032) nonwoven separators (Celgard® 5550) or anion exchange membranes (AEM; A201 by Tokuyama) were soaked in 4 mol dm^−3^ KOH solution and used as separator between the Zn foil or Zn sponge anode, respectively, and the GDE (10 mm diameter). Additional 25 µL of KOH solution (4 mol dm^−3^) were used to wet the exposed separator membrane surface again before placing the GDE. To limit parasite reactions such as OER occurring on cathode current collector, a titanium mesh (10 mm diameter, 0.076 mm wire thickness, Alfa Aesar) was used as current collector at the cathode side. As particular modification in the coin-type cell, a stainless steel spacer (15.5 mm diameter, 0.2 mm thick, MTI Corporation) was placed underneath the Sn current collector, and a stainless steel spring (15.4 mm diameter, 1.1 mm thick, MTI Corporation) was on top of the titanium mesh current collector. Finally, the coin cell was automatically closed with a crimping machine (MTI Corporation) at a pressure equivalent to 1 ton per coin cell area and inserted in sealed gas container (total volume of approximately 0.5 L, filled with humidified O_2_ (purity 5.0, Praxair).

**Fig. 1:**
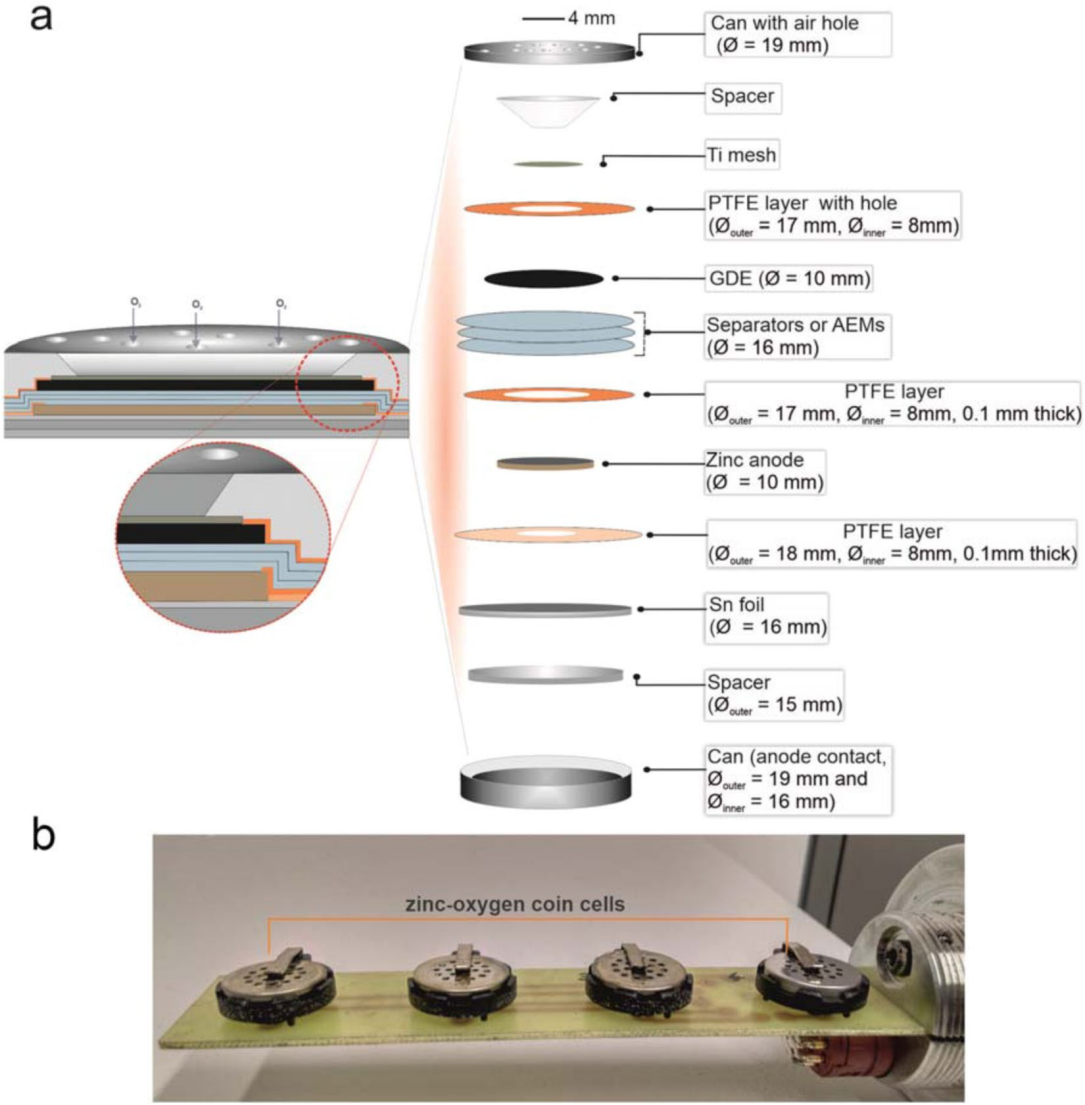
(**a**) Schematic of the cell assembly of the zinc oxygen battery coin-type cell. (**b**) Photograph of the electronic board with test cells that was placed inside the sealed gas container (containing humidified O_2_) during experiments. It can host a maximum of four cells.

Figure 1 provides information on the coin-type cell and Table 1 lists the cell components used: It is to be noted that CR2032 coin-type cells already contain one plastic sealing ring in the bottom can, which we make use of to achieve isolation. To further prevent infiltration of electrolyte in-between all void spaces, a thin flexible film layer made of polytetrafluoroethylene (PTFE) isolated the contact space between the stainless steel can, i.e. the current collector at the anode, and a spacer.

**Table 1:** List of components used in the coin-type cell assembly of the battery.

Components	Parameters
Anode active material	Zinc foil (12.0 mm diameter, 2.0 mm thickness) or zinc sponge (size defined by its theoretical capacity; compare^11^)
Anode current collector	Tin (Sn), 16.0 mm diameter, 125 μm thickness
Cathode active material	Humidified pure O_2_ at 10^5^ Pa, purity 5.0
Catalytic layer	Sr_2_CoO_3_Cl, RuSn73 or and Fe_0.1_Ni_0.9_Co_2_O_4_/xwt%AC on carbon support (gas diffusion layer, catalyst loading, binder and carbon support as used by Stock *et al*.^11^ as well as by You and Hu^12^, Lu *et al*.^13^ respectively)
Cathode current collector	Stainless steel spring, 15.4 mm diameter and 1.1 mm thick
Separator	Nonwoven separators (Celgard® 5550) or anion exchange membrane (A201 by Tokuyama), 16.0 mm diameter
Electrolyte	In total 175 µL of 4 M KOH(aq)

The inner diameter of the PTFE layer at the Sn foil at the Zn anode can be adjusted depending on the diameter of the anode. The space between the current collector and the Zn anode is isolated by using another thin layer of PTFE or Parafilm® (Bemis Company Inc; blend of 50% petroleum wax and 50% polyolefins). The size of the inner diameter of this PTFE layer is carefully chosen so that only the border of the Zn anode is not exposed to the aqueous alkaline electrolyte in the separators (see inset in Fig. 1). The contact area between the electrolyte and the current collector as well as the cathode can be minimized by placing another PTFE layer on top of the GDE.

### Measurement and data collection

Table 2 gives an overview of the herein applied experimental conditions for each battery cell.

**Table 2:** Summary of all experimental conditions for the cycled cells as labeled as in the data repository^15^.

Cathode 1	Cycling parameters	
	Discharge	Charge
Cell 1 – Cell 2 (Fig. 2)	−5.0 mA cm^−^², *t*_limit_ = 30 min	5.0 mA cm^−^², *t*_limit_ = 30 min
Cell 3 – Cell 7 (Fig. 3)	−2.0 mA cm^−^², *Q*_limit_ = 3% *Q*_theo_	2.0 mA cm^−^², *Q*_limit_ = 3% *Q*_theo_
Additional data 1 → Cell 8	−1.0 mA cm^−2^, *t*_limit_ = 1 h	1.0 mA cm^−2^, *t*_limit_ = 1 h
Additional data 1 → Cell 9	−5.0 mA cm^−2^, *Q*_limit_ = 15% *Q*_theo_	5.0 mA cm^−2^, *Q*_limit_ = 15% *Q*_theo_
Additional data 1 → Cell 10	−1.0 mA cm^−2^, *E*_limit_ = 0.9 V	1.0 mA cm^−2^, *Q*_limit_ = *Q*_discharge_
**Cathode 2**		
Cell 11 (Fig. 4)	−10 mA cm^−^², *t*_limit_ = 5 s	10 mA cm^−^², *t*_limit_ = 5 s
Additional data 2	0, −2, −10, −20, and −50 mA cm^−^², *t* = 10 min each step (ORR)	0, 2, 10, 20 and 50 mA cm^−^², *t* = 10 min each step (OER)
**Cathode 3**		
Additional data 3 → Cell 12 (Fe_0.1_Ni_0.9_Co_2_O_4 + _AC mixture)	−2, −5, −10, −20, −50 and −100 mA cm^−^², *t*_limit_ = 10 min	2, 5, 10, 20, 50 and 100 mA cm^−^², *t*_limit_ = 10 min
Additional data 3 → Cell 13 (Fe_0.1_Ni_0.9_Co_2_O_4_/3.7 wt%AC)	−2, −5, −10, −20, −50 and −100 mA cm^−^², *t*_limit_ = 10 min	2, 5, 10, 20, 50 and 100 mA cm^−^², *t*_limit_ = 10 min

*Q* is thereby the battery capacity in mAh, *t* is the time and *E* the potential.

**Fig. 2:**
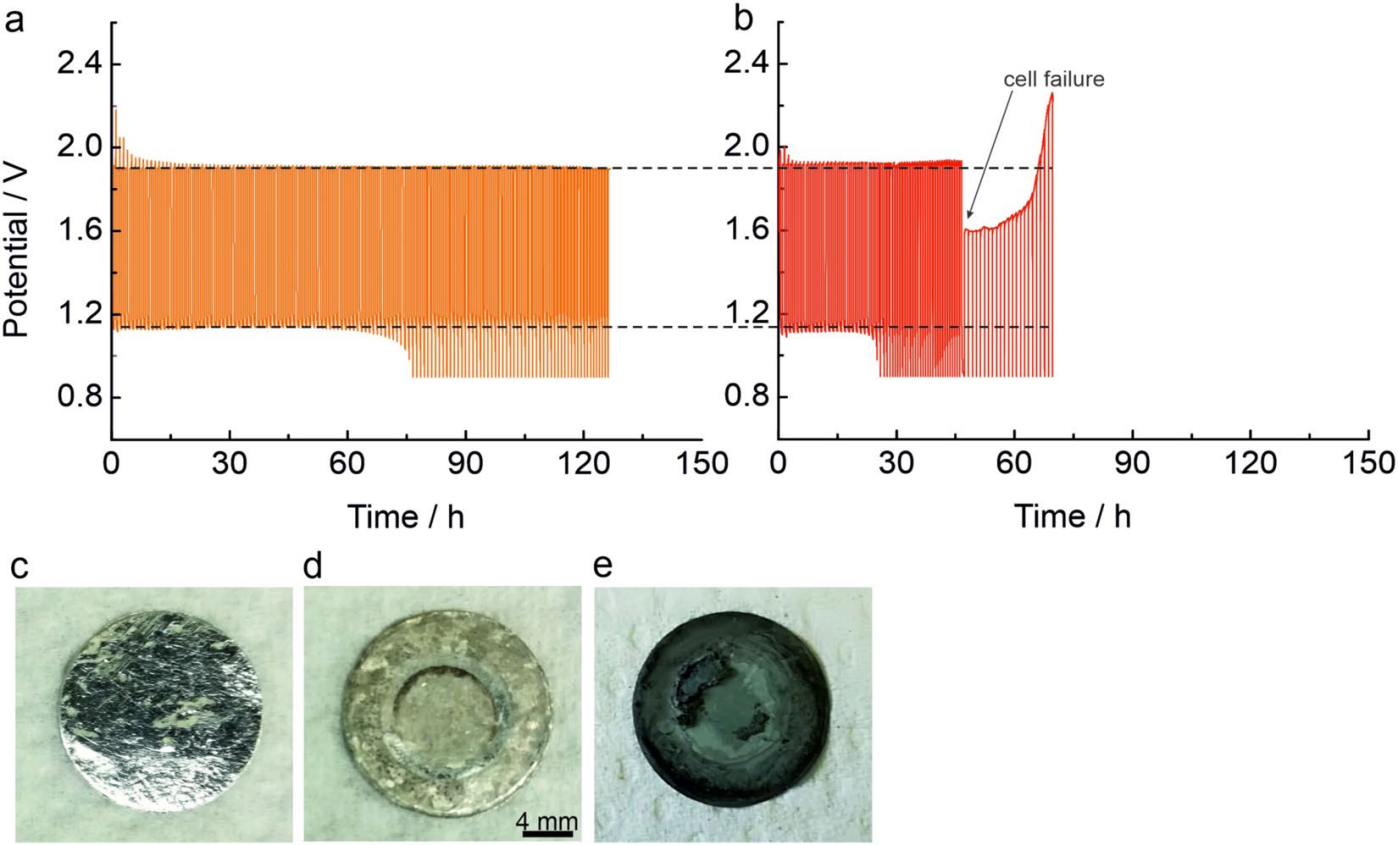
Cycling profile (30 min discharge and charge; 0.22% of the theoretical capacity based on the mass of Zn) of electrically rechargeable zinc oxygen batteries at 5.0 mA cm^−^² and Zn foil as anode with: (**a**) the spaces between housing, Sn current collector and zinc anode are carefully isolated with PTFE as depicted in Fig. 1; (**b**) the electrolyte can infiltrate the spaces in between because no isolation is used. The cell potential presents a significant change during discharge and charge when the electrolyte infiltrates the spaces in between the components, which allows side reactions to take place. Photographs of the Sn foil current collector taken from exemplary, comparable batteries: (**c)** pristine; (**d**) after cycling for 125 h at 2.0 mA cm^−^² with isolation between the spaces (**e**) after cycling for 70 h at 2.0 mA cm^−^² with electrolyte infiltration between spaces. Here, the RuSn73-GDE was used as cathode^12^ for all batteries.

**Fig. 3:**
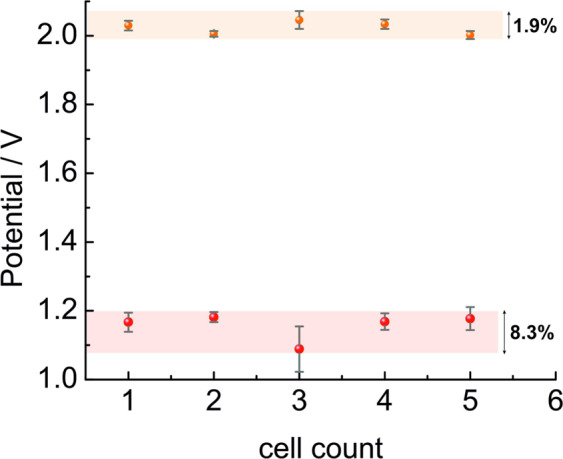
Averaged cell potentials of 30 discharge steps (bottom; red color) and 30 charge steps (top; orange color) for five as-prepared zinc oxygen batteries cycled at 2.0 mA cm^−^² and limited to 3% of the theoretical capacity. The cell potential only deviates marginally between the batteries. Here, a Zn sponge with anion exchange ionomer (AEI) was used as the anode^11^ and a Sr_2_CoO_3_Cl-GDE was used as the cathode^11^ for all batteries. The standard deviation in percent between the averaged cell potentials of each cell is given during charge and discharge. The errors bars indicate the standard deviation in cell potential during the course of cycling for each cell individually.

Electrochemical characterization for the results shown in Figs. 2 and 3 was performed using a SP-300 potentiostat/galvanostat (Biologic) and a BCS-805 battery cycler (Biologic) at room temperature. The data were collected every 5 seconds by the EC-Lab software from Biologic, version V11.16. The ZOB coin cells were cycled inside a sealed gas container (total volume of approximately 0.5 L, filled with humidified O_2_ (purity 5.0; Praxair)). The cells were cycled galvanostatically at different currents.

For additional data (see data repository), reproducibility measurements were performed at ±2.0 mA cm^−2^ during cycling, whereas the capacity was limited to 3% of the theoretical capacity (based on the weight of the active material; Q = 3% of Q_s_, where Q_s_ is the theoretical specific capacity of the Zn anode of 819 mAh g^−1^) for five coin cells. Shallow cycling experiments were performed at ±1.0 mA cm^−2^ for one hour per discharge/charge corresponding to 0.09% of the theoretical capacity, using a Zn foil with 12 mm diameter as anode. Full discharge and charge experiments with Zn sponge anode were performed at ±2.5 mA cm^−2^ with a cut-off voltage during discharge set to 0.9 V and the charge step limited to a capacity that was equal to the previous discharge capacity. Cell cycling experiments were also performed at ±5.0 mA cm^−2^ for approximately 15% of Q_s_ during each discharge/charge cycle with a Zn sponge anode. The Zn sponge anodes had a mass of approximately 3.56 mg.

Data gathered for the cycling at ±10.0 mA cm^−2^ of batteries with the RuSn73-GDE were recorded with the electrochemical analyzer CHI1128C and 730D (CH Instruments, Inc.), respectively; these data were collected every 5 seconds (and every 0.01666 min for the polarization test in the additional data in the repository) by the software provided by CH Instruments, Inc.

Batteries with GDEs of different loading of Fe_0.1_Ni_0.9_Co_2_O_4_/xwt%AC were cycled at various current densities providing data for cell 12 and 13. This data were recorded with the electrochemical analyzer CHI1128C (CH Instruments, Inc.).

## Data Records

The data are provided as txt files or as csv files^15^.

Each txt file provides output data and contains either the open circuit voltage (OCV), the discharge and charge profile of the battery during cycling with Cathode 1 (see Figs. 2 and 3 as well as additional data for other current densities in the repository^15^). The output contains various information, including: current (mA), voltage between anode and cathode (V), electrode potential of the GDE (V), capacity (mAh), and time (s) or (min), respectively, as summarized in Table 3. (It is to be noted that other important values to assess battery performance, such as energy (Wh), specific capacity (mAh g^−1^) or specific energy (mWh g^−1^), can be calculated with the experimental information and from the data provided if desired.)

**Table 3:** Metadata of open circuit voltage, discharge and charge response of the tested zinc oxygen batteries.

Value	Unit	Description
Cycle number	—	Number of the respective full cycle of discharge and charge; labeled as *cycle number*
Current	mA	Set current during measurement
Voltage	V	Measured voltage; labeled as *Ecell*
Electrode potential	V	Measured potential; labeled as *Ewe*
Capacity	mAh	Measured capacity of the battery; labeled as *Capacity* or *Q discharge* or *Q charge*
Time	s or min	Total operating time; labeled as *Time* or *time*

**Fig. 4:**
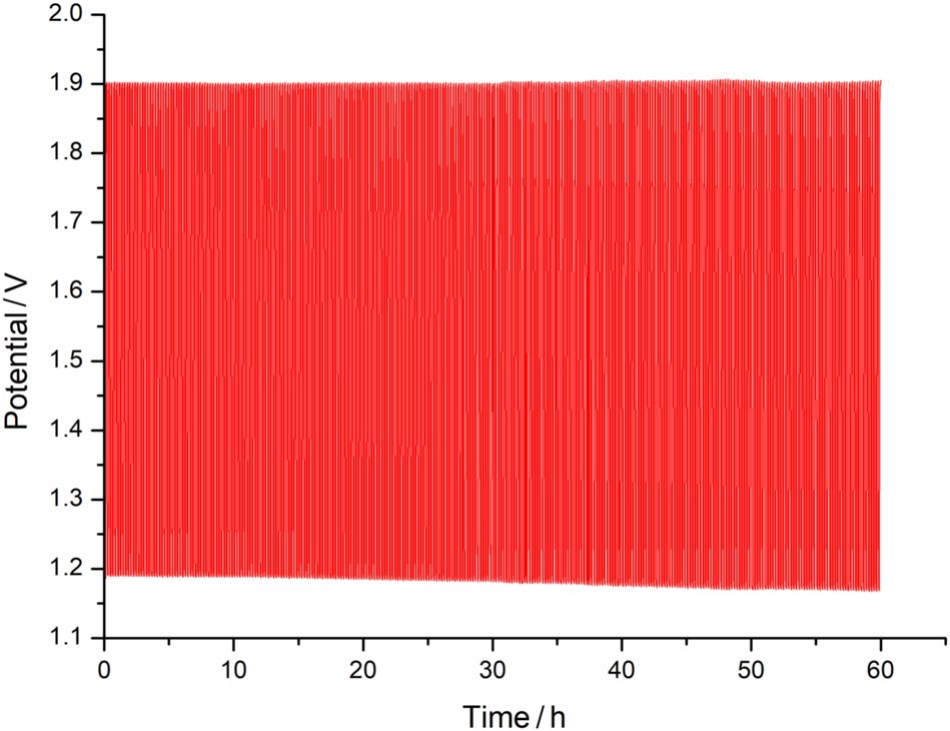
Cycling profile at 10.0 mA cm^−2^ of a test cell with the bifunctional catalyst RuSn73 at the cathode and Zn foil as the anode with almost now decay in the cell potential during charge and discharge. It is to be noted that a different setup is used than for the coin-cell data; see You and Hu^12^.

Each csv file provides the discharge and charge profiles of the battery during cycling with Cathode 2 (see Fig. 4) or the voltage responses during the ORR and OER for a step-wise increase of the current applied (see Figure 8 a and b in the work by You and Hu^12^, respectively).

The txt files for the additional data obtained with Cathode 3 (see Figure 8 in the work by Lu *et al*.^13^) provide the discharge and charge profiles of the battery during cycling for a step-wise increase of the current applied.

## Technical Validation

In the experiments, data were collected each time from freshly prepared batteries and data have not endured any other manipulation like the elimination of erroneous data points.

In a ZOB the aqueous alkaline electrolyte is needed at the anode, in the separator and at the cathode for the electrochemical reactions and for the transport of ions, respectively. During battery testing, the electrolyte might leak from the separator into all elements of the cell assembly where it can undergo severe side reactions with e.g. the housing, the spacer, the current collector^14^. Testing an as-prepared battery in coin-type geometry with PTFE isolation in place ensures that only the intended reactions and processes take place, which ensures at the same time that a stable and reproducible data set for the cycling profile, i.e. the cell potential during discharge / charge, can be recorded. It is for example well known, that side reactions of the electrolyte with cell components can have a direct impact on the cell potential – in particular for the evolution of hydrogen at the anode^16^ yielding a mixed potential.

The cell potential for a battery with PTFE isolation in the assembly and without is shown in Fig. 2a. Whereas the cell potential is a little below 1.9 V during charge for the battery with PTFE isolation, it is above 1.9 V for the battery without isolation. This electrochemical behavior indicates that side reactions take place yielding an increased overpotential. The photographs of the Sn foil current collector in pristine state (Fig. 2c) and after cycling (Fig. 2d,e) support this observation. The Sn foil current collector has changed its color to dark gray – presumably due to severe side reactions with the electrolyte – in the battery without PTFE isolation (Fig. 2e), whereas it preserved its color in the battery with PTFE isolation in place (Fig. 2d).

The reproducibility of the cycling data using the herein described assembly was tested for five different batteries for which we determined the cell potential during cycling for 60 h. Each data point in Fig. 3 is ascribed to one battery and shows its averaged potential values during charge and discharge, respectively. We observe a very small deviation between the cell potentials of the different batteries (about 8.3% maximum during discharge and 1.9% maximum during charge), which is an indicator that the data is reproducible. With the herein described assembly procedure for the battery test cell, possible sources for side reactions can be minimized and thus the variation of the cycling performance is minimized as well.

Exemplarily, the cycling stability of a GDE is documented by the performance with the bifunctional catalyst RuSn73 shown over 60 h (Fig. 4). The potential during discharge remains almost constant at 1.2 V whereas stable charge is maintained around 1.9 V.

## Usage Notes

The data described herein can be employed to validate the results obtained with theoretical models for electrically rechargeable zinc oxygen batteries. Besides, the additional data on the GDE performance in the data repository can be used to fit the parameters, e.g. for the kinetics of the oxygen reduction and oxygen evolution with bifunctional catalysts, of empirical models. Moreover, studies on the degradation of zinc oxygen batteries may use the data to implement the results on the long-term cycling to analyze degradation rates.

## Data Availability

Not applicable because the reported data were generated from experiments.
